# Impact of cognitive stimulation on ripples within human epileptic and non-epileptic hippocampus

**DOI:** 10.1186/s12868-015-0184-0

**Published:** 2015-07-25

**Authors:** Milan Brázdil, Jan Cimbálník, Robert Roman, Daniel J Shaw, Matt M Stead, Pavel Daniel, Pavel Jurák, Josef Halámek

**Affiliations:** Behavioural and Social Neuroscience Research Group, CEITEC-Central European Institute of Technology, Masaryk University, Brno, Czech Republic; Department of Neurology, Brno Epilepsy Center, St. Anne’s University Hospital and Medical Faculty of Masaryk University, Pekařská 53, Brno, 65691 Czech Republic; International Clinical Research Center, St. Anne’s University Hospital, Brno, Czech Republic; Department of Physiology, Medical Faculty of Masaryk University, Brno, Czech Republic; Department of Neurology, Mayo Systems Electrophysiology Laboratory, Mayo Clinic, Rochester, MN USA; Institute of Scientific Instruments, Academy of Sciences of the Czech Republic, Brno, Czech Republic

**Keywords:** High-frequency oscillations, Hippocampal ripples, Epilepsy, Human cognition

## Abstract

**Background:**

Until now there has been no way of distinguishing between physiological and epileptic hippocampal ripples in intracranial recordings. In the present study we addressed this by investigating the effect of cognitive stimulation on interictal high frequency oscillations in the ripple range (80–250 Hz) within epileptic (EH) and non-epileptic hippocampus (NH).

**Methods:**

We analyzed depth EEG recordings in 10 patients with intractable epilepsy, in whom hippocampal activity was recorded initially during quiet wakefulness and subsequently during a simple cognitive task. Using automated detection of ripples based on amplitude of the power envelope, we analyzed ripple rate (RR) in the cognitive and resting period, within EH and NH.

**Results:**

Compared to quiet wakefulness we observed a significant reduction of RR during cognitive stimulation in EH, while it remained statistically marginal in NH. Further, we investigated the direct impact of cognitive stimuli on ripples (i.e. immediately post-stimulus), which showed a transient statistically significant suppression of ripples in the first second after stimuli onset in NH only.

**Conclusion:**

Our results point to a differential reactivity of ripples within EH and NH to cognitive stimulation.

## Background

Transient high-frequency oscillations (HFOs) in the frequency range 80–250 Hz have been recorded repeatedly in animal and human hippocampi. These field potentials, referred to as “ripples”, were observed initially with microrecordings in rats and later in human hippocampi of epileptic patients [1–3]. Ripples are believed to reflect short-term synchronization of neuronal activity and appear to play important roles in both normal and pathological brain functions [4, 5]. More recently, strong evidence has been presented for a link between memory consolidation and hippocampal ripples in both micro- and macroelectrode recording studies [6, 7]; ripples related to the strengthening and reorganization of memory traces are observed during slow wave sleep and quiet wakefulness [7–10].

Importantly, ripples have also been observed in the epileptic dentate gyrus, wherein they are absent in healthy animals [4, 11–13]. It seems feasible, therefore, that a proportion of ripples recorded within the epileptic hippocampus are linked to underlying pathological processes. Indeed, clinical macroelectrode recordings within mesial temporal structures in epileptic patients inconsistently report an increase of ripples on the side of epileptogenic tissue. This contrasts with observations from previous microrecordings in animals [14]. This has led some to suggest that very large electrodes may filter out physiological HFOs, such that only pathological HFOs remain visible [15]. Even in macroelectrode studies, however, different electrode types might lead to differing results concerning the contribution of hippocampal ripples to the lateralization of an epileptogenic region. Furthermore, their clinical/diagnostic utility is compromised by the inability to distinguish normal from pathological ripples in invasive electroencephalographic (EEG) recordings. Achieving such a differentiation is important not only to understand the fundamental mechanisms behind normal cognitive functions, but also for the utilization of ripples as a potential clinical biomarker for the identification of an epileptogenic region.

The aim of the present study was to investigate a potential means with which to differentiate between physiological and epileptic ripples in intracranial recordings. Specifically, we tested our hypothesis that cognitive stimulation would have a differing effect on ripples within epileptic and non-epileptic hippocampi in human subjects. To do so, we analyzed hippocampal activity in ten epileptic patients during quiet wakefulness and during a cognitive task.

## Methods

### Subjects

Our sample comprised 10 patients (three males) ranging in age from 20 to 47 years (mean age: 31 ± 7.5 years), all with medically intractable focal epilepsies (Table 1). All subjects were on chronic anticonvulsant medication, reduced slightly in most cases for the purpose of video-EEG monitoring. All but one of the patients (no. 3) were right handed. Written informed consent was obtained from each subject prior to the investigation and the study received approval from the Ethics Committee of Masaryk University, Brno.

**Table 1:** Patient characteristics

Subject	Gender	Age at SEEG	FS	Age at Seizure onset	MRI before SEEG	Type and side of epilepsy	SOZ	Intervention/histopathology	Postoperative outcome (follow-up)	Number of analyzed hippocampal contacts
1	F	28	−	13	Meningoencephalocele and hypotrophy of left temporal pole	TLE/L	Left temporopolar region	AMTR and plastic surgery of skull base	Seizure-free (3 years)	3 (E)
2	F	23	−	13	Local atrophy and gliosis in the horn of left lateral ventricle after exstirpation of pilocystic astrocytoma within parieto-occipital region	ETLE/L	Left parieto-occipital bounder and occipital lobe	Reoperation: lesionectomy/reparative changes after exstirpation of astrocytoma	Seizure-free (1 year)	3 (N)
3	F	37	−	17	Normal	TLE/R	Right hippocampus	AMTR/FCD	Seizure-free (3 years)	8 (E)
4	M	35	−	23	Normal	TLE/R	Right hippocampus	AMTR/normal	Improved (2.5 years)	5 (E), 5 (N)
5	F	33	+	5	Normal	TLE/R	Right hippocampus and temp. pole	AMTR/FCD	Improved (1.5 years)	5 (E), 3 (N)
6	F	25	−	6	Susp. cortical dysplasia TPO left	ETLE/L	Left lateral tempo–parieto–occipital region	Focal cortectomy/normal	Not improved (2 years)	3 (N)
7	M	47	+	6	Slight atrophy of right hippocampus	ETLE/R	Right lateral parieto-occipital region	VNS/–	Non-responder (1.5 years)	7 (N)
8	M	35	−	28	MTS left	TLE/L	Left hippocampus	AMTR/gliosis	Improved (3 years)	2 (E), 6 (N)
9	F	28	−	3.5	normal	ETLE/R	Multifocal frontal	VNS/–	Responder (1 year)	3 (N)
10	F	20	−	16	Left temporal pole resection due to epilepsy	TLE/bilat	Left and right hippocampus (independ.)	VNS/–	Non-responder (1 year)	8 (E)

*SEEG* stereoelectroencephalography, *FS* febrile seizures, *SOZ* seizure onset zone, *MTS* mesiotemporal sclerosis, *TPO* temporo–parieto–occipital, *TLE* tempoval lobe epilepsy, *ETLE* extratemporal lobe epilepsy, *R* right, *L* left, *AMTR* anteromesial temporal resection, *VNS* vagal nerve stimulation, *FCD* focal cortical dysplasia, *(E)* epileptic, *(N)* non-epileptic.

### EEG recordings

Depth electrodes were implanted to localize seizure origin prior to surgical treatment. Each patient received 5–11 orthogonal platinum electrodes in the temporal and/or frontal, parietal, and occipital lobes using the stereotaxic coordinate system of Talairach [16]. Standard depth electrodes (ALCIS) were used with a diameter of 0.8 mm, a contact length of 2 mm, and an inter-contact distance of 1.5 mm. The exact positions of the electrode contacts in the brain were verified using postplacement MRI with electrodes in situ. The EEG signal was recorded with sampling rate of 1,024 Hz (TrueScan EEG system, Deymed Diagnostic). All recordings were referenced to a linked earlobe. All impedances were less than 5 kΩ. In this study, we investigated EEG data from 61 contacts positioned in epileptic (31) and non-epileptic (30) hippocampi (Table 1). This categorization was performed according to the results of standard visual analysis of ictal stereo EEG recordings; the seizure onset zone (SOZ) was defined as the contacts showing the first EEG ictal activity.

### Behavioral tasks

During the initial rest period, subjects were asked to relax for 30 min while sitting comfortably in a reclining position with eyes closed. For the subsequent cognitive phase they performed a visual oddball task. Stimuli consisted of capital letters presented randomly in the center of a monitor, with target (“X”) and frequent (“O”) trials intermixed with other distractor letters at a ratio of 1:4.6:1 (target:frequent:distractor). There were 50 target stimuli. The stimuli were presented for 500 ms, and the inter-stimulus interval varied between 4 and 6 s. Each subject was instructed to respond to the target stimulus as quickly as possible by pressing a button with their dominant hand.

### Data analysis and statistics

Using an automated detection of ripples based on the signal power envelope, we analyzed potential differences in ripple rate (RR) during the cognitive compared with the resting period, within epileptic (EH) and non-epileptic hippocampi (NH). Further, we compared the direct impact of cognitive processes on ripples (i.e. immediately post-stimulus) in EH and NH.

**Figure 1:**
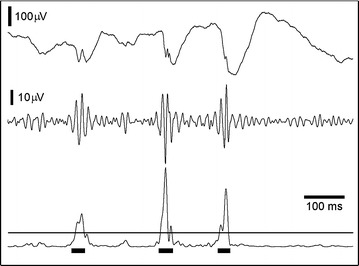
A demonstration of ripple detection. *Top* to *bottom* raw data from a single subject and contact; ripples within the signal filtered at 80–250 Hz; automated detection using amplitude of power envelope.

In the pre-detection stage the signal power envelope for 80 and 250 Hz band pass was calculated using the Hilbert transform. The HFOs were detected using normalized power envelope amplitude and duration thresholds (Figure 1). In order to stress the high power events and suppress the surrounding signal, the normalization of signal power envelopes (normPE) was performed by subtracting 2/3 percentile (p_66_) of the signal power envelope PE(f) and subsequently dividing it by half the value of the 2/3 and 1/3 percentile (p_33_) difference:

$$normPE = \frac{{PE(f) - p_{66} }}{{(p_{66} - p_{33} )/2}}$$

**Figure 2:**
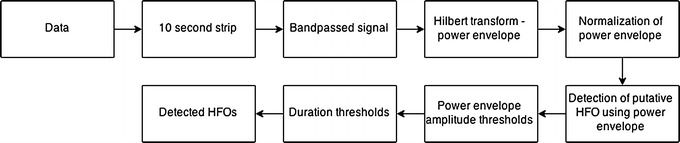
Block diagram of computational method for ripple detection.

The amplitude threshold values of normPE were based on normalized characteristics of HFOs that were scored previously by expert reviewers in various empirical data sets (Figures 1, 2). The duration threshold was set to a minimum duration of ~30 ms which was ~5 oscillations at 150 Hz.

To assess the effect of the cognitive stimuli, we examined the occurrence of ripples/RR for each contact in a moving window with the length of 0.5 s shifted in 0.05 s increments. The statistical significance of differences relative to baseline (−0.6 to −0.1 s pre-stimulus) was analyzed by Wilcoxon signed rank test.

We then investigated whether any differences existed in the duration of HFOs between EH and NH, during both resting-state and cognitive stimulation. As the durations of HFOs varied according to a normal distribution, we used unpaired t test to identify any significant differences.

To distinguish between ripples and high-frequency activities (HFAs), the latter of which appear to be related to the multiunit firing rate (e.g. task-induced gamma), we further generated time frequency maps (TFM) and power envelopes averaged to trigger stimuli in the frequency range 80–250 Hz. TFM provides an overview of the time–frequency increase in gamma activity associated with stimuli, and power envelopes enable comparisons between induced gamma power and power of ripples. These computations were performed on the signals from all hippocampal contacts and montages.

## Results

**Figure 3:**
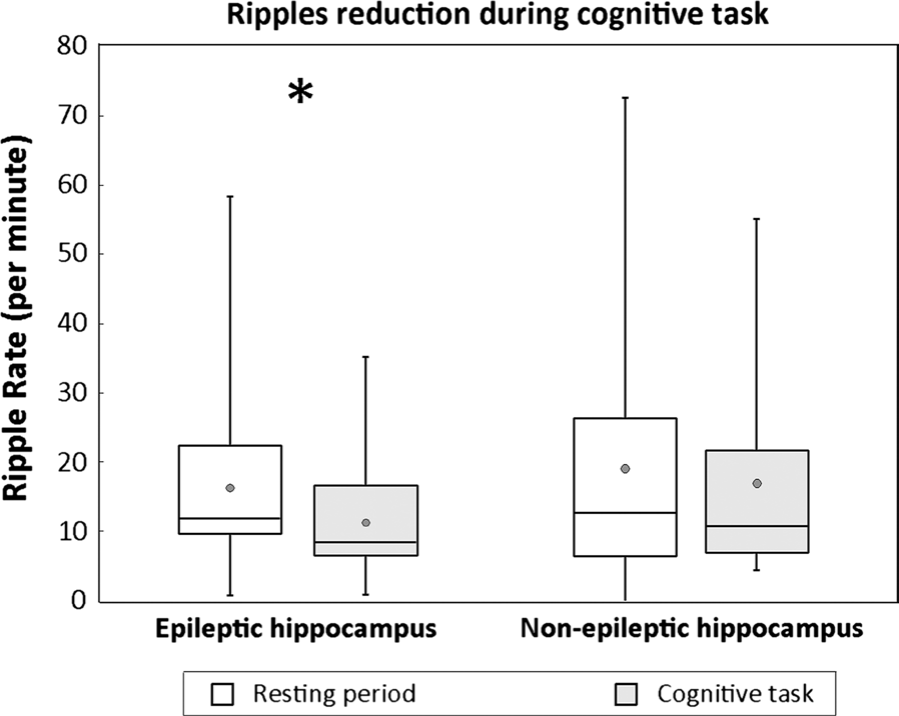
Ripple rates during resting-state and cognitive-task periods within epileptic and non-epileptic hippocampi across all investigated subjects. *Black asterisk* means significant difference in epileptic hippocampus (p < 0.05).

Ripples were detected within hippocampal recordings from all subjects. Mean RR (across all EH or NH contacts) in resting-state periods was 16.4/min [SD = 12.2; range 1–58] within EH, and 19.4/min [SD = 17.4; range 0–73] within NH. Over cognitive-task periods, mean RR within EH and NH decreased to 11.2/min [SD = 7.7; range 1–35] and 17.2 [SD = 15.0; range 5–55], respectively (Figure 3). Median and quantile [0.1/0.9] values were as follows: resting-state EH 11.9 [2.7/32.6]; resting-state NH 12.8 [5.4/41.5]; cognitive task EH 8.4 [3.1/22.6]; cognitive task NH 10.9 [5.6/47.7]. The reduction of mean RR during the execution of cognitive task was significant in EH (p < 0.001) but only marginal in NH (p = 0.06; indicating a trend). There was no significant difference in RR within EH versus NH during the resting state, but a significant difference was revealed by statistical analysis of RR between EH and NH during cognitive task performance (p < 0.05).

**Figure 4:**
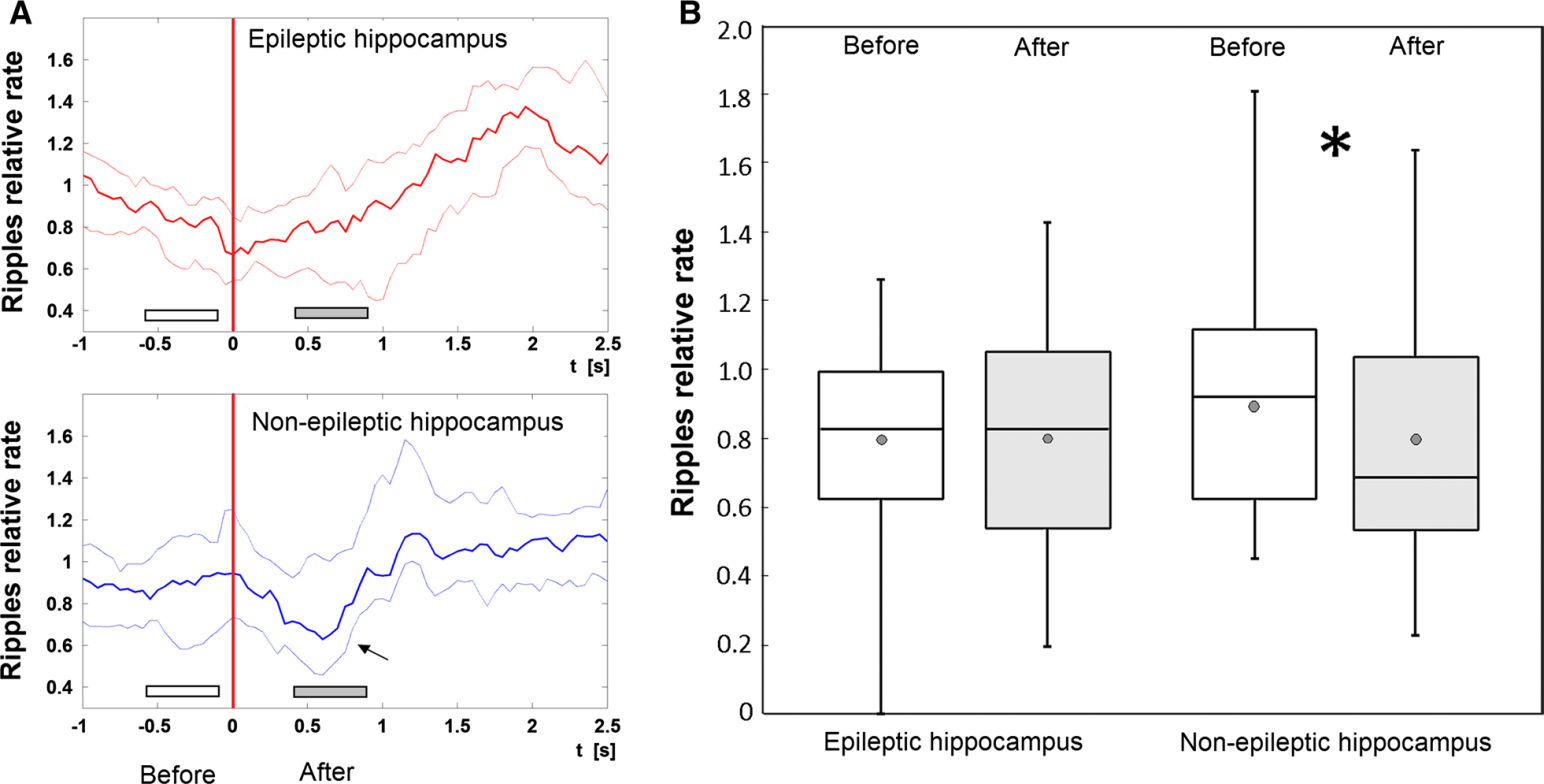
Immediate short-lasting impact of cognitive stimuli on ripple rate across subjects. **a** Transient suppression of relative ripple rate within epileptic (*upper* **a**) and non-epileptic (*bottom* **a**) hippocampus. *Red vertical line* defines visual stimulation onset (trigger). *Full lines* represent median, *dotted lines* 25 and 75 percentile across all subjects and all recording contacts. The figure clearly demonstrates task-induced HFOs reduction in non-epileptic hippocampus in time period approximately 0.3–1 s after the stimulation (*arrow*). *White* and *gray horizontal bars* indicate an area that corresponds to the *box plots* in the *right* **b**. **b** *Box plots* computed in baseline period before stimuli (−0.6 to −0.1 s) and after cognitive stimulation (0.4–0.9 s). *Black asterisk* means significant difference in non-epileptic hippocampus (p < 0.02).

**Figure 5:**
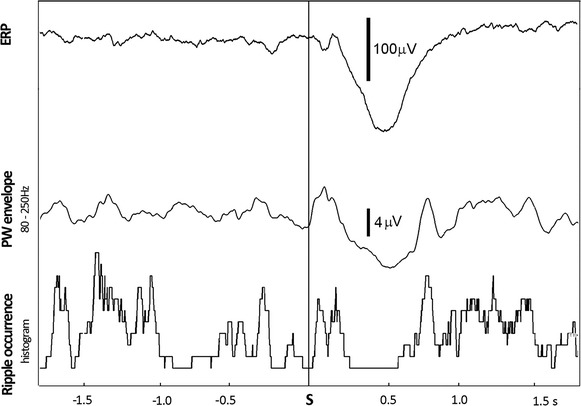
Observations from a single contact within the non-epileptic hippocampus of subject (patient no. 6). *Bottom* to *top* the transient post-stimulus decrease in ripple occurrence within non-epileptic hippocampus coincides with event related 80–250 Hz power envelope reduction, the genesis of local-field cognitive potential (P3). *S* stimulus.

Investigating the immediate impact of cognitive stimuli on ripple occurrence, we observed a statistically significant short-lasting suppression of ripples in NH within the first second after stimuli onset. This effect was virtually missing in EH (Table 2; Figures 4, 5). Interestingly, the short-lasting suppression in NH was followed by a robust and significant transient increase in RR approximately one second after the stimuli. A similar but somewhat later significant RR increase was also observed in EH (Figure 4a).

**Table 2:** Changes in relative ripple rates immediately after cognitive stimul ation (computed in 500 ms moving window, significant changes compared to baseline −0.6 s to −0.1 s are highlighted)

Epileptic hippocampus													
Time (s)	Baseline	0–0.5	0.1–0.6	0.2–0.7	0.3–0.8	0.4–0.9	0.5–1.0	0.6–1.1	0.7–1.2	0.9–1.4	1.0–1.5	*1.1*–*1.6*	*1.3*–*1.8*
Mean RR	0.7911	0.7595	0.7445	0.7630	0.8172	0.8130	0.7939	0.7842	0.8262	0.8951	0.9374	*1.0221*	*1.1213*
SD	0.2896	0.2174	0.2584	0.2941	0.3187	0.3711	0.3240	0.3025	0.3405	0.3934	0.3821	0.3699	0.3173
P	1.0000	0.3916	0.1524	0.4799	0.6655	1.0000	0.6718	0.5682	0.2026	0.3123	0.0854	*0.0293*	*0.0094*
Non-epileptic hippocampus													
Time (s)	Baseline	0–0.5	0.1–0.6	0.2–0.7	0.3–0.8	*0.4*–*0.9*	*0.5*–*1.0*	0.6–1.1	0.7–1.2	*0.9*–*1.4*	*1.0*–*1.5*	1.1–1.6	1.3–1.8
Mean RR	0.8880	0.9033	0.8405	0.7830	0.7782	*0.7443*	*0.7943*	0.9045	1.0418	*1.1370*	*1.3300*	1.1489	1.0960
SD	0.2845	0.3465	0.3754	0.3893	0.3795	0.3705	0.3754	0.3501	0.4175	0.4225	0.3969	0.3886	0.3059
P	1.0000	1.0000	1.0000	0.6778	0.3317	*0.0127*	*0.0055*	0.6778	0.3222	*0.0127*	*0.0110*	0.0919	0.6778

The mean duration of ripples in resting-state periods was 88.1 ms [SD = 7.4 ms; range 73.8–150.6 ms; median = 87.9 ms; 10% quantile = 77.8 ms] within EH, and 82.8 ms [SD = 9.8 ms; 61.0–109.2 ms; median = 81.2 ms, 10% quantile = 71.8 ms] within NH. In cognitive-task periods, mean ripple duration (RD) within EH and NH were 85.5 (SD = 5.2 ms; range 71.9–98.8 ms; median = 85.9 ms; 10% quantile = 80.1 ms] and 83 (SD = 10.1 ms; range 63.0–100.6 ms; median 85.9 ms, 10% quantile = 70.0 ms], respectively. There was a significant difference between EH and NH ripple durations during quiet wakefulness (p < 0.001), but we did not observe any significant EH/NH difference for RD under the cognitive load (p = 0.136). The changes in ripple duration during cognitive stimulation did not reach statistical significance for either EH (p = 0.052) nor NH (p = 0.902), although a trend was observed in case of EH.

Individual TFM analyses of signals from all the investigated non-epileptic hippocampal sites revealed unequivocal task-induced gamma (HFA) in 6 of 30 channels (3 subjects in total), and only after targets. The frequency range of this gamma activity overlapped partially with ripple activity. However, averaged TFM and power envelopes reveal that the power of induced gamma on single trials was significantly lower than the threshold for ripple detection. This significant difference in power excludes the false detection of induced gamma activity as ripples.

## Discussion

In the present study we have used simple cognitive task to investigate whether the effect of cognitive stimulation on hippocampal ripples can be used as a new approach for distinguishing presumably normal HFOs in NH from presumably pathological HFOs in EH. We observed significantly different, and in some aspects opposite, behavior of ripples within EH and NH. We must stress that we have not explored the nature of ripples in both NH or EH in the present experiment; our finding of different HFO content/dynamics in the response of the epileptic and non-epileptic hippocampus to cognitive stimulation offers little insight into the difference between physiological and pathological ripples. On the other hand there is a consensus to support that ripples are not only normal activity in hippocampus. Many reports from humans and non-primates showed an increase in ripple frequency HFO at, or prior to, the onset of seizures [17]. It seems unlikely that these “pathological” ripples reflect just an “exaggerated” version of physiological activity as suggested recently by [6]. Rather, while physiological hippocampal ripples (and underlying sharp wave-ripple/SPWR/complexes) appear to reflect summated synchronous inhibitory postsynaptic potentials generated by subsets of interneurons regulating the discharges of principal cells [1], epileptic HFOs represent field potentials of population spikes from clusters of abnormal synchronously bursting neurons [4, 18, 19]. Recent cellular evidence suggests that physiological ripple oscillations reflect phasic perisomatic inhibitory synaptic potentials in pyramidal cells, together with sparse phase-locked firing and rhythmic depolarizing potentials [20–22]. Inhibitory interneurons could then secure an orderly recruitment of pyramidal cells [23, 24]. Pathological ripple-like high-frequency oscillations might represent heterogeneous cellular and synaptic phenomena. The recent study of Alvarado-Rojas et al. revealed an involvement of distinct synaptic processes and different mechanisms of synchrony in the origin of ripple-like HFOs (150–250 Hz) during interictal (IID) and preictal epileptic discharges (PID). IID ripples were associated with rhythmic inhibitory postsynaptic potentials and weak phase-locked firing, whilst PID were associated with depolarizing potentials that usually triggered rhythmic burst firing [21]. Both types of pathological ripples must be distinguished from fast ripples in the 250–800 Hz range, which reflect population spikes of partially synchronous, massively bursting, uninhibited pyramidal cells [22]. Regardless, the present findings of different ripple dynamics to external stimulation seem to suggest diverse mechanisms behind their generation.

The question remains whether the mechanisms of generation, as well as the functional significance of waking- and sleep-related ripples (both normal and pathological), are similar or different. Unfortunately, sleep related HFOs have been studied much more extensively in the past, with less evidence coming from ripple studies performed during quiet wakefulness. A growing number of papers have, however, focused on awake SPWRs, suggesting their relationship to behavioral performance and complementary role in memory consolidation with sleep SPWRs [7, 9, 25–28]. It was shown that physiological as well as pathological hippocampal HFOs can be recorded reliably with standard macroelectrodes in awake periods, and it is interestingly that the occurrence of ripples changes as a function of the state of vigilance. However, the results of previous micro- and macroelectrode studies on memory are contradictory; using microelectrodes recordings in rodents, monkeys, and humans, ripples appear typically to be more present during immobility and slow-wave sleep [1, 29, 30]. On the other hand investigating HFOs from human non-epileptic hippocampus during a memory consolidation task and using macroelectrodes recordings, [7] report that the majority of all hippocampal ripples occurred during waking state, with only a minority occurring during stages of deep sleep. In contrast, epileptic ripples are significantly more frequent during non-REM sleep compared to epochs of wakefulness in both micro- and macrorecordings [31].

Evaluating ripple occurrence in resting and task periods, our study revealed a significant decrease of HFO rate in epileptic tissue during event discrimination. This finding might reflect an increased involvement of hippocampal neurons in physiological cognitive processing, and consequently decreased synchronization within the network driven by synchronously bursting epileptic neurons. This hypothesis seems to be congruent with the well-known prevalence of epileptic ripples during non-REM sleep (see above), which results very likely from the sleep-dependent enhancement of network synchronization within the mesial aspect of the temporal lobe [32–34]. Our observation that cognitive stimulation only marginally impact upon general ripple rate within the non-epileptic hippocampi can be explained by anticipated involvement of normal hippocampal neurons in both memory consolidation/awake neuronal replay (rest period) and complex event discrimination processing (task period). Finally, the oddball task used in our study is linked intimately with an information-processing cascade, during which attentional and memory mechanisms are engaged preferentially [35].

In contrast to the significant effect of event processing on long-standing RR within epileptic hippocampi and only marginal effect in presumably healthy structures, we observed an immediate short-lasting impact of cognitive stimuli on ripple occurrence in non-epileptic hippocampi only. Specifically, we reveal that single cognitive stimuli appear to suppress NH ripples briefly. Despite difference in experimental paradigms, this finding might be analogous to the selective suppression of SPWRs by timed electrical stimulation [36, 37]. Noteworthy, in a study published recently [38], no disruption of SPWRs was observed during the presentation of simple non-cognitive light stimuli in adult rabbits. The question thus remains whether or not simple sensory stimulation is sufficiently potent to disrupt physiological hippocampal ripples in human macrorecordings. In our study this immediate effect took approximately 500 ms and culminated in the peaking of an averaged event-related potential known as P3, which consists largely of contributions from theta and delta oscillations (Figure 5). We can therefore assume a compromising of the activity of normal hippocampal neurons between distinct physiological processes, resulting in either high frequency or slow oscillations. Finally, the subsequent transient increase in ripple occurrence approximately 1 s after stimulus onset might represent a simple rebound of HFOs or possibly true increase of ripples, that was recently observed in human hippocampi approximately 800 ms after the presentation of typical visual memory stimuli [41]. Theoretically it may also reflect some task-induced high-frequency neural activity (HFA), which seems to be related largely to multi-unit activity (MUA) and could be linked to memory consolidation and ongoing retrieval of stored memories [8, 10, 39, 40]. In our study, however, the methodology used for ripple identification prevented the false detection of task-induced gamma; averaged power envelopes revealed very different powers in ripples and gamma activities, the latter of which was significantly lower than the threshold level for ripple detection. Particularly noteworthy is our observation of task-induced gamma in only a minority of hippocampal contacts and subjects in this study. This might be somewhat surprising given that the hippocampus is likely to be engaged in some memory processes during the oddball task [35]. On the other hand, this limited occurrence of task-induced gamma in hippocampal regions after target stimuli might explain the small increase in the blood oxygen level-dependent (BOLD) signal (in which gamma is reflected) within these regions in functional MRI studies using the oddball task [42]. Finally, task-induced gamma occurs usually between 100 and 400 ms after stimuli presentation, and is therefore likely to precede ripple suppression [43]. Regardless, the more limited direct impact of external stimuli on ripple rate within epileptic hippocampi likely reveals the low reactivity of epileptic neurons to physiological events.

Distinguishing normal and pathological HFOs represents currently one of the most challenging tasks for basic and clinical neuroscience. Pathological HFOs may have important diagnostic and prognostic value, serving potentially as biomarkers for the epileptogenic zone that is a crucial target for highly effective epilepsy surgery. Research on normal HFOs can then give us a better understanding of memory encoding and consolidation, as well as better insight into the distortion of these processes in neurocognitive disorders. To the best of our knowledge this is the first study providing evidence of significantly different effects of external sensory stimulation on ripples within epileptic and non-epileptic hippocampi. Until now only one published paper has addressed specifically the differentiation of normal and pathological HFOs. Using visual or motor task in five epileptic patients, distinct parameters of two types HFOs have been suggested [43]. The identification of normal and pathological HFOs according to frequency content is currently a matter of intense experimental scrutiny, and certainly cannot be used within the ripple range [44]. Despite previous suggestions of the distinct filtering of normal HFOs by very large electrodes, this effect is highly equivocal [14]. Even in our present study we found slightly (but not significantly) higher RR in non-epileptic compared to epileptic hippocampi, a result that clearly contradicts the proposed “filtering” effect. No other approaches to reach this “holy grail” for hippocampal HFOs are available today.

An obvious limitation of our study is the analyses of data from chronic epileptic patients only. Even if we carefully differentiated epileptic and non-epileptic hippocampi with presumed epileptic and normal ripples, the impact of epileptic activity on normal neurons even within non-epileptic hippocampi cannot be excluded completely. This risk is higher when treating contralateral hippocampus in patients suffering from unilateral temporal lobe epilepsy. In our study, this risk was reduced because the majority of non-epileptic hippocampal recordings were taken from extratemporal epilepsy patients. Still, translating our “normal” results into normal hippocampus behavior must be done with great caution. On the other hand, intracerebral EEG recordings are possible only in patients suffering from major brain disorders. As such, this limitation must be kept in mind.

## Conclusions

In conclusion, the results of our study point to a differential reactivity of ripples recorded from within EH and NH to cognitive stimulation. This discovery could present a possible means with which to identify hippocampal epileptic ripples in laboratories that record intracerebral EEG data with the standard sampling frequency of 1 kHz.
